# *Notes from the Field:* Respiratory Viral Panel as an Early Diagnostic Tool for Neonatal Enterovirus Infection — San Diego, California 2023

**DOI:** 10.15585/mmwr.mm7327a2

**Published:** 2024-07-11

**Authors:** Ryan Sanchez, Errica Capossela, Mark Speziale, Jane O’Donnell, Amaran Moodley, Christina Morales, Debra A. Wadford, Carol Glaser, Seema Shah, Mark E. Beatty, Alice Pong

**Affiliations:** ^1^Division of Neonatology, Department of Pediatrics, University of California San Diego, San Diego, California; ^2^Rady Children’s Hospital-San Diego, San Diego, California; ^3^Division of Pediatric Infectious Diseases, Department of Pediatrics, University of California San Diego, San Diego, California; ^4^Viral and Rickettsial Disease Laboratory, Center for Laboratory Sciences, California Department of Public Health; ^5^Center for Laboratory Sciences, California Department of Public Health; ^6^County of San Diego Health and Human Services Agency, San Diego, California.

**Summary**

**What is already known about this topic?**

Enterovirus infections can cause severe disease in neonates.

**What is added by this report?**

In 2023, a cluster of neonatal enterovirus infections initially suspected to be echovirus 11, but subsequently identified as Coxsackie B4 and B5 infections, occurred in San Diego, California. Respiratory panel polymerase chain reaction (PCR) testing for rhinovirus-enterovirus facilitated diagnosis of enterovirus infection in these infants.

**What are the implications for public health practice?**

Coxsackie virus as well as echovirus can cause severe disease in neonates. Respiratory virus panel PCR testing in neonates can be a useful diagnostic tool for enterovirus sepsis evaluations.

Enterovirus infections in neonates can result in high morbidity and mortality. In 2023, a cluster of neonatal enterovirus cases associated with Coxsackie B4 and B5 occurred in San Diego, California.

## Investigation and Outcomes

During June–October 2023, five cases of neonatal enterovirus infection were identified at Rady Children’s Hospital in San Diego, California. The authors were granted a waiver of individual authorization for use of protected health information by the University of California San Diego Institutional Review Board. All five cases were initially suspected to be caused by enterovirus based on characteristic clinical presentations during enterovirus seasons and supported by positive rhinovirus-enterovirus (RhV-EV) results from respiratory virus panel (RVP) testing of nasopharyngeal specimens, performed within 1 day of symptom onset. Reports of severe neonatal enterovirus disease from Europe linked to a new variant of echovirus 11 caused concern (*1*). Four of the five patients’ plasma also tested positive for enterovirus by reverse transcription−polymerase chain reaction (RT-PCR). Results of RT-PCR testing of cerebrospinal fluid (CSF) for enterovirus were positive for two patients. Four infants had thrombocytopenia, and three had hepatitis with coagulopathy. Serum ferritin levels were elevated in three neonates. One neonate experienced seizures as the initial sign and subsequently developed pancytopenia with suspected, but unconfirmed, viral-induced hemophagocytic lymphohistiocytosis. The most severely affected patient, an infant aged 5 days, whose mother experienced a febrile illness during delivery diagnosed as chorioamnionitis, developed multiorgan failure. The infant received multiple immune globulin intravenous (IGIV) doses, the investigational antiviral drug pocapavir* (*2*), and maternal convalescent plasma; however, the infant did not survive. Four of the five infants received IGIV therapy. Mothers of three of the infants received a diagnosis of chorioamnionitis before delivery, and the mother of the remaining two infants (twins) was reportedly evaluated for postpartum fever and received a diagnosis of endometritis. 

Blood, CSF, and respiratory specimens were sent to the California Department of Public Health Center for Laboratory Sciences Viral and Rickettsial Disease Laboratory for virus identification,^†^ in coordination with the County of San Diego. Coxsackie B5 was identified in three specimens and Coxsackie B4 in one. Plasma from the fifth patient tested positive for enterovirus by RT-PCR; however, the viral copy number was too low for further identification (Table).

**TABLE:** Pertinent laboratory values* and clinical features of neonatal patients with enterovirus infection — San Diego, California, 2023

Characteristic	Patient				
	A	B	C	D	E
**Sex**	Male	Male	Female	Male	Female
**Age at symptom onset, days**	9	6	5	6	5
**Gestational age, wks**	36	36	38	40	35
**Initial symptoms**	Respiratory distress and poor feeding	Poor feeding and seizures	Poor feeding	Fever and respiratory distress	Poor feeding and decreased tone
**AST / ALT (U/L)**	104 / 17^†^	970 / 367^§^	1,608 / 318^§^	69 / 26^†^	4,215 / 564^¶^
**Ferritin (ng/mL)**	Not done	112,984^§^	98,999^§^	Not done	>100,000^¶^
**Platelet count (× 1,000/*μ*L)**	64^§^	6^§^	20^§^	227^†^	8^¶^
**PT / INR ratio**	Not done	33.6 / 3.1^§^	18.9 / 1.5^§^	Not done	60.3 / 5.3^¶^
**Antiviral therapy**	None	IGIV	IGIV	IGIV	IGIV, pocapavir, MCP
**Outcome**	Survived	Survived, seizures	Survived	Survived	Deceased
**EV type identified**	Coxsackie B5	Coxsackie B5	Coxsackie B4	Copy number too low for detection	Coxsackie B5

**Abbreviations:** ALT = alanine transaminase; AST = aspartate aminotransferase; EV = enterovirus; IGIV = immunoglobulin intravenous; INR = international normalized ratio; MCP = maternal convalescent plasma; PT = prothrombin time.

* The most abnormal values identified for each category are presented; laboratory testing for patients A, D, and E was performed at different hospitals.

^†^ Reference values: AST = 17–184 U/L; ALT not established for age ≤28 days; platelet count = 150,000–450,000/*μ*L.

^§^ Reference values: AST = 32–162 U/L; ALT = 5–33 U/L; ferritin = 100–717 ng/mL (age 0−14 days), 14–647 ng/mL (age 15 days−6 months); platelet count = 140,000–440,000/*μ*L; PT = 12.3–15.3 sec; INR = 0.86–1.14.

^¶^ Reference values: AST = 0–32 U/L; ALT = 0–33 U/L; ferritin = 150–973 ng/mL; platelet count = 220,000–450,000/*μ*L (age 0−7 days), 230,000-600,000/*µ*l (age 8 days−6 months); PT = 9.7–12.5 sec; INR = no reference values for patients not receiving anticoagulation therapy.

## Preliminary Conclusions and Actions

Enterovirus infection can be life-threatening in the early neonatal period. At the time of this cluster, countries in Europe were reporting echovirus 11 infection in neonates (*1*). Coxsackie B4 and B5, but not echovirus 11, were identified among the patients described in this report. In a review of clinical characteristics of severe neonatal enterovirus infections during 2000–2020 (*3*), in cases where virus serotype was known, 82.7% of cases resulted from Coxsackie B viruses and 16.7% from echoviruses. Data from the U.S. National Enterovirus Surveillance System (*4*) showed that Coxsackie B viruses and echoviruses were also the most common groups of enteroviruses reported among U.S. neonates.

Maternal illness is reported in association with neonatal enterovirus infection and is a likely source of infection for infants with early illness (*3*,*5*). Details of maternal symptoms in this cluster of patients were not available for review; however, symptoms attributed to chorioamnionitis and endometritis might also have been due to maternal enterovirus infection.

Detection of rhinovirus-enterovirus among the five patients at illness onset, despite absence of upper respiratory tract symptoms, led to high suspicion of enterovirus and thus a targeted neonatal sepsis workup. Timely RVP testing is not always performed for neonates. If rapid on-site enterovirus-specific RT-PCR testing is not available, including nasopharyngeal RVP testing as part of the neonatal sepsis workup, particularly during summer and fall, could facilitate diagnosis of neonatal enterovirus infection. Timely identification facilitates optimal clinical management for the infant, which might include receipt of IGIV and possibly antiviral medication.

## Footnotes

* Pocapavir was obtained through ViroDefense, Inc. and the Food and Drug Administration expanded access program.

†
https://journals.asm.org/doi/10.1128/jcm.00542-06
